# Associations between sleep bruxism and (peri-)implant complications: lessons learned from a clinical study

**DOI:** 10.1038/s41405-020-0028-6

**Published:** 2020-01-30

**Authors:** Magdalini Thymi, Corine M. Visscher, Daniel Wismeijer, Frank Lobbezoo

**Affiliations:** 1 Department of Orofacial Pain & Dysfunction, Academic Centre for Dentistry Amsterdam (ACTA), University of Amsterdam and Vrije Universiteit Amsterdam, Amsterdam, The Netherlands; 2 Department of Oral Implantology and Prosthetic Dentistry, Academic Centre for Dentistry Amsterdam (ACTA), University of Amsterdam and Vrije Universiteit Amsterdam, Amsterdam, The Netherlands

**Keywords:** Peri-implantitis, Dental equipment, Fixed prosthodontics

## Abstract

**Objective:**

To report and discuss the lessons learned from the conduct of a clinical study on the associations between sleep bruxism and (peri-)implant complications, the protocol of which has been pre-published.

**Materials and methods:**

A single-center, double-blind, prospective cohort study with a 2 year follow-up was performed in the Academic Centre for Dentistry Amsterdam (ACTA), The Netherlands. Eleven adult participants were included, where an inclusion of 98 was planned. Sleep bruxism was assessed by multiple single-channel electromyographic (EMG) recordings. Main outcomes were biological and technical complications. Results of the study are presented alongside with comments on encountered difficulties.

**Results:**

Insufficient participant recruitment and failed EMG recordings were encountered. The small sample size did not allow answering the study’s main aim, and was mainly attributed to the study’s protocol complexity. EMG recording failures were attributed to insufficient quality of the EMG signal and detachments of the electrode.

**Discussion:**

The lessons learned from the conduct of this study can be used to design successful future clinical studies.

**Conclusions:**

Adequate participant recruitment, effective EMG recordings, and a careful selection of predictor variables are important ingredients for the successful conduct of a longitudinal clinical study on the association between sleep bruxism and (peri-)implant complications.

## Introduction

Bruxism, either sleep or awake, can be a significant source of overload for dental implants. As such, its association with (peri-)implant complications is already hypothesized for a long period of time.^1^ The body of literature on this topic has been growing steadily in the past few years. Systematic reviews have shown a positive association between bruxism and implant failures.^2–4^ However, as pointed out in these reviews, the results need to be taken with great caution, since the reviewed studies suffer from poor bruxism definitions and a lack of objective methods to diagnose bruxism.^2–4^ More recently, a prospective cohort study using an objective diagnostic method for sleep bruxism, i.e., single-night portable electromyography (EMG), found no relationship between high intensity of sleep bruxism and a higher risk of complications in patients with implant-supported fixed complete dentures.^5^

To our knowledge, no other prospective studies designed to address bruxism as a risk factor for dental implant complications have been published, nor are there any studies on this topic registered in major public trial registries, i.e., clinicaltrials.org and clinicaltrialsregister.eu (search terms bruxism AND implant, date of search Oct 28, 2019). Ideally, studies on the topic should comply with the following:

- Appropriate case definition. This includes a clear reference to which bruxism definition has been used, and a distinction between sleep and awake bruxism. Assessment of sleep bruxism should at least include an objective instrumental assessment, such as (ambulatory) polysomnography (PSG) or EMG recordings. Variability in sleep bruxism should be taken into account, and this would correspond to the need for multiple overnight recordings. Awake bruxism should ideally be addressed by instrumental means,^6^ and in the absence of those, a standardized questionnaire can be used.^7^
- Appropriate outcome definition. Clearly defined biological and technical complications,^8^ and inclusion of patient-reported outcome measures (PROMs), i.e., esthetic outcomes and measures that reflect the patients’ perception of their implant treatment success.^9^
- Adequate sample size. Sufficient size to allow for sound statistical analyses, even in the case of rare outcomes, e.g., implant fractures. Analyses should take collinearity into account that arises from the fact that multiple implants can be present in the mouth of a single participant.^10^

In 2017, our research group published the protocol of a study that complies with nearly all these features.^11^ The primary aim of this study was to investigate whether sleep bruxism is a risk factor for dental implant biological and technical complications. The secondary aim was to investigate whether there is an association between sleep bruxism and the composition of peri-implant submucosal biofilm. The study was approved by the local Medical Ethical Committee (Vrije Universiteit Amsterdam, ref.: 2011–245) in December 2014, and was registered in the Netherlands Trial Register (Trialregister.nl, ref.:4930) as well as by the US National Institutes of Health (ClinicalTrials.gov identifier: NCT02410681). Implementation of the study protocol took place between February 2015 and May 2019.

There were several important obstacles which hampered the conduct of the study when following the published protocol. As a result, our primary and secondary aims could not be answered. However, through the failure of adhering to the original study protocol, important lessons were learned. These can be used to promote the design of future, more successful research, with optimal utilization of human and financial resources.^12^ Taking this into account, reporting on the execution of the current study is of value to the scientific community.^12–14^ We are aware of the fact that this is not common in our field. Therefore, the purpose of this paper is to report and discuss the lessons learned from the conduct of a clinical study on the associations between sleep bruxism and (peri-)implant complications and not, in this case, the clinical results.

## Materials and methods

A comprehensive description of the study protocol is presented elsewhere.^11^ In short, a single-center, double-blind, prospective cohort study with a 2-year follow-up period was designed. The follow-up period initially consisted of eight visits, i.e., at baseline, 2 weeks, 6 weeks, 3 months, 6 months, 1 year, 18 months, and 2 years. Due to the low participation rates, two visits were omitted 8 months after the study was initiated, viz., the ones at 6 months and 18 months, in order to make the study protocol less burdensome (see “results” section) for the participants. Thus, the final study protocol consisted of six visits. Participants were recruited from the clinic of the Department of Oral Implantology and Prosthetic Dentistry of ACTA. Inclusion criteria were: planned treatment with implant-supported fixed suprastructure(s) and age ≥18 years. Exclusion criteria were: opposing teeth of implant-supported fixed suprastructure(s) are restored with removable artificial teeth; patients categorized in the classes 3 or higher according to the American Society of Anesthesiologists system for classification of physical status;^15^ use of an occlusal splint, mandibular repositioning appliance, or any other bruxism-mitigating device during sleep; active periodontitis at the time of implant placement; known allergy to the EMG device electrode material; usage of a pacemaker; and swollen, infected, or inflamed tissues or skin eruptions in the placement area of the EMG device electrode. Pregnancy after the placement of implants was not a reason to stop participation in the study. The aimed sample size was 98, as calculated by the formula: *n* = 50 + 8*k*; where k = the number of predictors, which was set at 6.^16^ Aimed duration of the study was 3 years: 1 year for sampling and 2 years for follow-up. Participants were compensated for their time at the amount of 60 euro upon completion of the follow-up period.

The primary predictor of the study was sleep bruxism, as assessed by the EMG activity of the right temporal muscle during sleep, measured with an ambulatory, single-channel EMG device (GrindCare, version 3 + DL, Delta Danish Electronics, Light and Acoustics, Hørsholm, Denmark). Besides recording EMG activity, the device can issue electrical impulses to lower the EMG activity. This feature was turned off before the device was given to the participants. Three sessions of overnight recordings, each consisting of three consecutive nights, were performed at baseline, 6 weeks, and 1 year follow-up. Quality of raw EMG data was assessed based on the following criteria: the presence of an acceptable signal-to-noise ratio (SNR), i.e., maximum voluntary contraction (MVC) amplitude >10 times the noise amplitude, during a sufficient length of the recording, i.e., at least 75% of the length of the recording, and absence of artefacts, such as detachment of the electrode. Participants were required to perform three MVCs in the first 30 min of each recording in order to enable subsequent scoring of bruxism episodes. A sticker reminding participants of this necessity was placed on all EMG devices. Scoring of bruxism events was performed according to published criteria, based on a 20% MVC threshold.^17^ The number of bruxism episodes per hour of sleep (Epi/h) and the bruxism time index (BTI, i.e., the total time spent bruxing divided by the total sleep time, times 100%) were derived per recording.

The main outcomes were biological and technical complications, and composition of peri-implant submucosal biofilm. Data on confounding and/or interacting variables were collected, i.e., smoking status, awake bruxism, peri-implant plaque accumulation, and periodontal parameters (i.e., number of clinical pockets with probing depths of ≥5 mm and bleeding index). Furthermore, for the complete description of our sample, data on morphological and restorative aspects of participants’ implant treatments were collected. A comprehensive overview of all collected data is provided in Table 1.

**Table 1:** Overview of study variables and data collection methods.

Type of variable			Scale	Instrument	Time point
Outcomes	Biological complications	Bleeding on probing	Modified gingival index (REF) (0: no bleeding when a periodontal probe is passed along the gingival margin adjacent the implant; 1: isolated bleeding spots visible; 2: blood forms a confluent red line on margin; 3: heavy or profuse bleeding)	Periodontal probe (Click-Probe 3/5/7/10 blue, KerrHave, Bioggio, Switzerland)	T1–T6
		Probing depth	Distance (mm) from the mucosal margin to the bottom of the deepest clinical probing site on the mesial, distal, buccal, and lingual sides of the implant. Per implant, the mean value of those sides is calculated		
		Marginal bone height	Vertical distance (mm) from implant shoulder to superior border of marginal bone in mesial and distal sides; the difference in marginal bone height between subsequent radiographs is calculated	Image acquisition: Vertical bitewing radiographs, parallel cone technique, phosphor plates (VistaScan Image Plate, Dürr Dental, Bietigheim-Bissingen, Germany), and individually putty-modified plate positioning devices Image processing: Emago software (version 6.0), Oral Diagnostic Systems, Amsterdam, The Netherlands	
		Loss of osseointegration	Presence/absence	Clinical and radiographic evaluation	
	Technical complications	Suprastructure	Complete or incomplete fracture of veneer, fracture of framework, loosening of occlusal screw or fracture of luting cement, fracture of occlusal screw	Clinical and radiographic evaluation	T3, T4, T5, T6
		Abutment	Loosening or fracture of abutment screw, fracture of abutment		
		Implant body	Fracture		
		Other	E.g., appearance of wear facets, loss of occlusal screw seal, etc.		
	Biofilm composition		Shannon diversity index of peri-implant submucosal biofilm, collected from the mesial, distal, lingual, and buccal sides of each implant	Biofilm collection and storage: Sterile paper points (Henry Schein Absorbent Points #504 medium, Henry Schein, Melville, NY, USA), tubes (Axygen Self-standing, clear, sterile Scientific Screw Cap Tubes, 2.0 ml, Axygen, Union City, CA, USA) and stored in the laboratory of ACTA at −80 °C Biofilm analysis: genome analysis of bacterial samples, open-ended sequencing technique	T1, T4, T5
Covariates/confounders	Smoking status		Never, occasional, former, current	Questionnaire (Hukkinen et al. REF)	T1, T5
	Awake bruxism		Never, rarely, sometimes, often, always	Questionnaire (Oral behaviors Checklist REF)	
	Plaque accumulation		Modified plaque index (REF) (0: no detection of plaque; 1: plaque only recognized by running a periodontal probe across the smooth marginal surface of the implant. Implants covered by titanium spray in this area always score 1; 2: plaque can be seen by the naked eye; 3: abundance of soft matter)	Clinical examination	T1, T2, T5
	Periodontal parameters		Number of clinical pockets with probing depths of ≥5 mm and bleeding index of all present natural elements and implants (except implants under study)	Periodontal probe (Click-Probe 3/5/7/10 blue, KerrHave, Bioggio, Switzerland)	
Sample descriptive variables	Occluding pairs of natural teeth		Number of pairs between upper and lower equivalent natural teeth	Clinical examination	T1, T5
	Implant geometrical characteristics		Type of implant (manufacturer and system), implant size (diameter and length, in mm)	Electronic health record	T1
	Implant loading characteristics		Time of loading with the definitive suprastructure (immediate, i.e., within 1 week after implant placement; early, i.e., between 1 week and 2 months; or late, i.e., after 2 months), bone or soft tissue augmentation procedures, bone quality (REF), and position of implant (within arch, lower or upper jaw)	Electronic health record	T1
	Prosthetic characteristics		Type of abutment (material, fabrication method), type of implant-supported suprastructure (single crown, fixed partial denture with or without cantilever), retention type (cemented or screw-retained)	Electronic health record	T2
	Antagonist status		Structure of opposing occlusal contact(s) (natural tooth, implant or none), type of restorative material present on opposing supporting cusp(s), and occlusal contact of implant-supported restoration with antagonists during maximum intercuspation, protrusion, and/or laterotrusions	Electronic health record and clinical examination with a 12 μm occlusal foil (Hanel occlusion foil 12 μm, Coltene, Langenau, Germany)	T2, T5

T1: baseline (impression appointment), T2: 2 weeks (suprastructure placement), T3: 6 weeks, T4: 3 months, T5: 1 year, T6: 2 years.

## Results

In order to serve the ‘lessons learned’ purpose of the current article, results on the topics of participant recruitment, sample characteristics, and sleep bruxism recordings are presented together with comments on encountered difficulties. Apart from the abovementioned issues, the measures taken to deal with these difficulties are discussed, giving this results section a mixed results-discussion format. Furthermore, descriptives for the main outcome variables are provided. A general discussion of the results will be presented in the last section of this article.

### Participant recruitment

Participants were recruited in the clinic of the Department of Oral Implantology and Prosthetic Dentistry of ACTA between February 2015 and August 2016 by one of the authors (MT). The recruitment procedure is described elsewhere,^11^ and is presented in more detail here. Eligible patients were first approached by their treating clinician right after their visit to the clinic for a regular appointment, such as postoperative evaluation of healing of the implanted site, and asked whether they woud be interested to receive more information about the study. If positive, they were thoroughly informed about the study: patients were given verbal explanation of the study’s procedures, followed by a written information letter. By combining the information procedure with a regular visit to the clinic, the burden for eligible patients was kept to a minimum. Hereafter, patients were given 1-week time to consider participation. They were contacted by phone or during their next regular visit to the clinic to confirm participation. Informed consent was signed prior to the first study-related visit.

Recruitment took place during the clinic’s operating hours, viz., two and a half days of the week, during 48 weeks of the year. Per week, nine clinicians each spent 0.3 full-time equivalents (FTEs) treating patients. Approximately eight patients are seen per 0.2 FTE. Thus, the clinic operates for a total of 2.7 FTE per week, in which ~8 patients are seen. Based on these numbers, the capacity of the clinic was deemed sufficient for the study’s purposes. During the recruitment period, 39 individuals fulfilled the inclusion and exclusion criteria. Of those, 28 (72%) were not included in the study due to the following reasons: participation in another study (*n* = 1), not willing to participate (including not willing to be informed about the study) (*n* = 16), unable to contact after initial screening (*n* = 6), and planned future use of an occlusal splint after initial screening, as proposed in the final prosthetic treatment plan (*n* = 5). Thus, 11 participants were included in the study.

#### Comment

There was a low number of individuals fulfilling the inclusion and exclusion criteria, and from among those, very few agreed to participate in the study. Those who declined participation (*n* = 16) were not required to provide a reason; however, some voluntarily did. Verbally reported reasons were: not having enough time for extra visits, not willing to sleep with a device in fear of receiving radiation, and, most importantly, not willing to commit to a lengthy obligation after having gone through an intensive implantological treatment trajectory. In order to tackle the issue of low participation, it was decided, 8 months after recruitment was started, to decrease the participant burden of the study protocol by omitting two study visits. Furthermore, the recruitment period was, within the limits of the study’s budget, extended by 7 months. The omission of two study visits did not have a positive effect on the inclusion rate, thus, after this period, it was decided to terminate recruitment. Upon approval of the local medical ethics committee, the study continued to complete the follow-up of already included participants, as to provide pilot data for the design of future studies. All participants were informed about this decision and were free to continue or terminate their participation in the study. No participants terminated their participation on these grounds.

### Sample characteristics

Eleven participants (three females), with a mean (s.d.; range) age of 54.8 (9.8; 32–66) were included in the study. The total number of implants was 19. Eight participants (2 females, 13 implants) completed the 1-year follow-up period, and six of them (1 female, 9 implants) completed the entire study. Reasons for dropping out were: not willing to perform more EMG recordings (*n* = 1), suprastructure not placed (*n* = 1), planning of occlusal splint due to suspected parafunction after inclusion (*n* = 1), and unable to contact for future appointments (*n* = 2). Descriptives of the sample that completed at least the 1-year follow-up are presented in Table 2.

**Table 2:** Sample characteristics (*n* = 8 participants, who completed at least 1-year follow-up).

	T1; Baseline (*n* = 8 participants; 13 implants)		T2; 2 weeks (*n* = 7 participants; 11 implants)	T5; 1 year (*n* = 8 participants; 13 implants)	
Smoking status	Never: 2 Occasional: 1	Current: 0 Former: 5	–	Never: 3 Occasional: 1	Current: 0 Former: 4
Awake bruxism	Never: 4 Rarely: 1 Sometimes: 3	Often: 0 Always: 0	–	Never: 1 Rarely: 5 Sometimes: 2	Often: 0 Always: 0
Modified plaque index^a^	0 (0–0)		0 (0–2)	0 (0–0)	
No. of pockets ≥5 mm	<2: 7 ≥2: 1		<2: 6 ≥2: 0	<2: 8 ≥2: 0	
Bleeding Index (%)^a^	10 (7–16.25)		6 (3.75–13.75)	5 (3.5–7.75)	
Occluding pairs of natural teeth^a^	9.5 (7.25–11.5)		–	8 (7.25–9.75)	
Implant manufacturer and system	Straumann SP RN Roxold SLActive: 4 Straumann BL NC Roxolid SLActive: 5 Straumann BL RC Roxolid SLActive: 4		–	–	
Implant size^a^	Diameter: 4.1 (3.3–4.1) Length: 10 (10–12)		–	–	
Time of loading	Late: 13		–	–	
Augmentation (soft and/or hard tissues)	No: 5 Yes: 8		–	–	
Implant position	Upper: 10 Lower: 3	Anterior: 6 Posterior: 7	–	–	
Type of suprastructure	Single crown: 9 Fixed partial denture (no cantilever): 4		–	–	
Type of abutment	Titanium prefabricated: 5 Titanium custom made: 8		–	–	
Type of retention	Cemented: 4 Screwed: 9		–	–	
Suprastructure material	Metal: 1 Full ceramic: 10 Metal-ceramic: 2		–	–	
Opposing occlusal contact	–		Natural tooth: 11 Implant: 2	Natural tooth: 11 Implant: 2	
Contact in maximum intercuspation	–		No: 7 Yes: 6	No: 8 Yes: 5	
Contact during latero-/protrusion	–		No: 10 Yes: 3	No: 8 Yes: 5	

^a^Median (25th–75th percentile).

### Sleep bruxism recordings

In the total sample (*n* = 11), 94 overnight recordings were performed. Of those, 44 (47%) fulfilled the pre-established quality criteria. The remaining 50 recordings that could not be used presented the following issues: no MVC in the first 30 min of the recording (*n* = 22), low SNR (*n* = 8), electrical pulses accidentally turned on (*n* = 5), detachment of the electrode (*n* = 12), and recording performed but not stored on the SD card (*n* = 3). In the sample that completed the 1-year follow-up (*n* = 8), a total of 79 recordings were performed, of which 40 (51%) fulfilled the quality criteria. Issues in the insufficient 39 recordings were: no MVC in the first 30 min of the recording (*n* = 18), low SNR (*n* = 8), detachment of the electrode (*n* = 10), and recording performed but not stored on the SD card (*n* = 3). The characteristics of accepted recordings are presented in Table 3. There was no significant difference between different time-points for either episodes/h or BTI (repeated measures ANOVA, *F* = 0.554, *p* = 0.512, and *F* = 0.249, *p* = 0.787, respectively).

**Table 3:** Characteristics of accepted recordings for participants that completed 1-year follow-up.

	T1	T3	T5
Duration (hour)^a^	6.07 (1.55)	6.61 (1.48)	6.05 (1.13)
Episodes/hour^a^	2.17 (1.4)	2.81 (2.31)	3.86 (2.56)
BTI^a^	0.95 (1.15)	0.46 (0.32)	0.59 (0.38)

^a^Mean (s.d.), T1: baseline; 8 participants/15 recordings, T3: 6 weeks; 5 participants/12 recordings, T5: 1 year; 6 participants/13 recordings, BTI: bruxism time index.

#### Comment

As seen from these data, the absence of MVCs, low SNR, and detachment of the electrode were the most important reasons for recording failures according to our quality criteria. Detachment of the electrode is a complication that will render an unusable recording, especially if it occurs early on in the recording. The issues of absent MVCs and low SNR might be tackled by alternatively scoring the EMG signal based on the times-noise-level method,^18^ i.e., by using the multiplication (e.g., two or three times) of the background EMG noise level as the event threshold. However, thus far, there is no consensus regarding the ideal scoring criteria of EMG signals acquired from ambulatory EMG devices,^19^ and deciding to adopt any alternative scoring method might thus be premature.

### Main outcomes

#### Biological complications

Profuse bleeding on probing, i.e., modified gingival index (mGI) = 3, was scored in two implants of a single participant on 3-month follow-up. All other bleeding on probing scores were low, with a median mGI of 0 at T1, T2, T4, and T6, and 1 at T3 and T5. Probing depths were small, and not indicative of clinical attachment loss. There were no clinically significant changes in marginal bone height. None of the implants showed mobility or were lost (Table 4a, b). Furthermore, at T6, two participants (three implants) reported sensitivity in the region of the implant.

**Table 4a:** Biological and technical implant complications in the 2-year follow-up period (participant level).

	T1; Baseline (*n* = 8)	T2; 2 weeks (*n* = 7)	T3; 6 weeks (*n* = 6)	T4; 3 months (*n* = 5)	T5; 1 year (*n* = 8)	T6; 2 years (*n* = 6)
Modified gingival index^a^	0 (0–2)	0 (0–2)	1 (0–2)	0 (0–3)	1 (0–2)	0 (0–2)
Probing depth^b^	1.8 (1–2.3)	1.3 (1–2.8)	1.5 (1.2–2.3)	2.8 (1.1–3.3)	1.4 (1–2)	1.8 (1.4–2.5))
Marginal bone height^b^	0.7 (0.4–1.4)	0.7 (0.5–1.5)	0.8 (0.4–1.6)	0.7 (0.6–1.8)	0.8 (0.2–1.4)	0.4 (−0.2 to 1)
Technical complications	–	–	0	2 (loosening of occlusal screw)	2 (occlusal wear)	3 (loosening of occlusal screw^1^, occlusal wear^3^)
Loss of osseointegration	0	0	0	0	0	0

^a^Median (minimum–maximum).

^b^Median (25th–75thq).

**Table 4b:** Biological and technical implant complications in the 2-year follow-up period (implant level).

	T1; Baseline (*n* = 13)	T2; 2 weeks (*n* = 11)	T3; 6 weeks (*n* = 9)	T4; 3 months (*n* = 10)	T5; 1 year (*n* = 13)	T6; 2 years (*n* = 9)
Modified gingival index^a^	0 (0–2)	0 (0–2)	1 (0–2)	0 (0–3)	1 (0–2)	0 (0–2)
Probing depth^b^	2 (1.3–2.3)	1 (1–1.8)	1.5 (1.1–2.3)	2.4 (1.3–3.3)	1.8 (1–2.1)	1.8 (1.3–2.5)
Marginal bone height^b^	0.7 (0.4–1.7)	0.9 (0.2–2)	1 (0.3–1.7)	0.9 (0.4–2)	0.9 (0.3–1.9)	0.3 (1–1.5)
Technical complications	–	–	0	2 (loosening of occlusal screw)	2 (occlusal wear)	5 (loosening of occlusal screw^1^, occlusal wear^4^)
Loss of osseointegration	0	0	0	0	0	0

^a^Median (minimum–maximum).

^b^Median (25th–75thq).

#### Comment

No significant issues related to the collection of biological data were encountered, with the minor exception of radiographic data acquisition in the anterior region. Though modified, the vertical bitewing positioning devices did not fit in this region. Alternatively, periapical plate positioning devices were used.

#### Technical complications

During the 1-year follow-up period, four technical complications were observed in four participants. At T4, a loosened occlusal screw occurred in two out of ten implants, in two participants. The appearance of wear facets was the most frequent complication. At T5, wear facets appeared in two out of eight implants, in two participants. At T6, wear facets appeared in four out of nine implants, in three participants. There were no issues related to the collection of technical complications data.

#### Biofilm composition

Analysis of collected biofilm data would not serve any meaningful purpose due to the small sample size, thus, it was not performed.

## Discussion

Longitudinal cohort studies using instrumental assessments of sleep bruxism on bruxism-complication-related topics, such as temporomandibular disorders^20^ and tooth wear^21^ are, to the best of our knowledge, absent in either published, or unpublished^22^ form. It is interesting to speculate on the reason behind the absence of such studies in a research field that otherwise receives ample attention. The inherent difficulty of conducting such studies may explain this research gap, and the results of our ‘failed’ study are supporting this notion. The purpose of this paper is to report and discuss the lessons learned from a clinical study on the associations between sleep bruxism and (peri-)implant complications., in order to promote the design of more successful future research. The execution of the pre-published study protocol was hampered by a number of issues, the most important of which were related to participant recruitment and the performance of sleep bruxism recordings.

It was not possible to achieve the predefined sample size (*n* = 98). Instead, in the 19 months that were available for participant recruitment, only 11 individuals enrolled in the study. Reasons for the low inclusion were a low number of individuals fulfilling the inclusion and exclusion criteria (*n* = 39), and, among those eligible individuals, a high noninclusion rate (*n* = 28, 72%). Not willing to participate was the most common reason for not including an otherwise eligible person (57% of noninclusions), and this issue was not adequately tackled when extra study visits were omitted from the protocol. Due to medical ethical considerations, it was not possible to demand and record a reason for non-participation. However, a number of individuals declining participation spontaneously provided this reason, and the matter was additionally discussed with the clinicians who were active in the clinic where the recruitment took place. From this, it could be concluded that deciding to commit to a longitudinal observational study that included multiple sleep registrations was felt as a burdening obligation, after a lengthy implant-related treatment. Individuals receiving dental implants often have gone through a long period of dental treatment of teeth that were eventually lost, before getting into implant treatment trajectories. However, prospective cohort studies are not uncommon in the field of oral implantology (e.g., see^8,23^). Therefore the tremendous difficulty in finding individuals willing to participate was not anticipated. In contrast to other prospective studies, though, the current study required active participant engagement, viz., multiple overnight recordings, which may have set the threshold for participation too high. It might also be hypothesized that this threshold could lead to selection bias. We could possibly attract participants who were already aware of sleep bruxism activity, and interested in objectifying this, and/or attract highly motivated individuals who wished to keep their implants healthy. These participants may be more interested in participating in a study with a strict follow-up regimen, with close monitoring of their implants. Furthermore, in this study, no differences were found for the sleep bruxism variables, i.e., Epi/h and BTI, between different time points in the course of 1 year, which could suggest that multiple recordings are not necessary. However, this finding should be taken with great caution, since the study sample was too small to draw any robust conclusion on the course of sleep bruxism over time. Variability in sleep bruxism activity in short^24^ and longer^25^ periods of time has been shown in other studies. More research on the natural course of sleep bruxism in needed,^7^ and in the meantime, addressing the time-variant nature of sleep bruxism through multiple sleep recordings at different time point is of importance in future studies.

Furthermore, even if all eligible individuals (*n* = 39) had agreed to enroll in the study, the sample size calculated would still have not been achieved in the amount of time permitted by the study budget. As described in the “Participant recruitment” section of the “Results”, based on the capacity of the Clinic of Oral Implantology and Prosthetic Dentistry of ACTA, no issues were expected related to the number of individuals fulfilling the inclusion and exclusion criteria. However, reality showed a high number of ineligible individuals due to removable prostheses in the opposite jaw, and/or (planned) wearing of occlusal splints after the implant/prosthetic treatment. The issue of low participation in a prospective cohort study with multiple overnight recordings and a lengthy follow-up period could be addressed by designing a multicenter study and/or allowing for a longer recruitment period. This decision should be based on thorough evaluation of the recruitment potential during the design phase of the study, and may have extensive financial and practical implications. The assessment of the recruitment potential in this study was based on calculations of the number of patients attending the clinic. The abovementioned issue of patients not willing to participate in the study might have been foreseen if patients had actively been involved in the research design phase, for example, through the conduct of interviews or panel discussions. Researchers should take into consideration that, depending on local law, such active involvement may require prior medical ethical approval. Active patient involvement was not applied in the design of the current study, since, as discussed above, the difficulties in participant recruitment were not expected. However, this method should be considered in similar future studies, or if there is any doubt on whether the recruitment process is sufficient.

The use of occlusal splints raises another issue that affects participant inclusion and can lead to selection bias. Occlusal splints are recommended by clinicians in the case that sleep bruxism is suspected, for example due to a history of severe dental attrition or repeated fractures of dental restorations.^26^ Thus, by using this as an exclusion criterion, it is possible that a high-risk group of bruxers is filtered out from the study sample, consequently biasing study outcomes. As shown in the retrospective study by Chrcanovic et al., “possible” and “probable” sleep and/or awake bruxism may be associated with an increased risk of dental implant failure.^27^ It would be very interesting to prospectively study such groups of bruxers with instrumental diagnostic devices. However, from a medical ethical point of view, conducting a study in which possible or probable sleep bruxers are not provided with an occlusal splint would be challenging, and perhaps not acceptable. Alternatively, patients wearing occlusal splints can be included in relevant studies, when the variable of wearing a splint would be taken into account in the statistical analysis.

EMG recording failures were mostly attributed to the absence of MVCs, low SNR, and detachment of the electrode. Detachment of the EMG device’s electrode from the skin and subsequent failure of the EMG recording has also been reported in other studies using the same device (e.g.,^28–30^) as well as for other ambulatory EMG devices (e.g.,^31^). Reasons for detachment might include insufficient cleaning of the skin prior to electrode placement, secretions of sweat and sebaceous glands during sleep, improper placement of the electrode, accidental pulling of the electrode wire, and electrode adhesive properties. To some extent, these factors can be addressed by using wireless electrodes, by improving electrode adhesive properties, and by providing participants with skin cleansing products for use prior to electrode placement.

A 20% MVC threshold was used in this study to score EMG events.^17^ Subsequently, the presence of at least one MVC was required in each recording, as well as a sufficient SNR. To this end, a ‘MVC amplitude >ten times the noise amplitude’ criterion was used. Unfortunately, MVCs were absent in 18 out of 79 (23%) recordings, despite thorough the instruction of participants and reminders placed on the devices. A similar problem is not frequently reported in literature. Failure of obtaining a MVC was reported for only 1 out of 108 participants in the study by Takaoka et al.^30^ Although compliance issues, such as forgetting, or not being able to use the device have been reported elsewhere (e.g.,^28^, it is not clear why participants in the present study did not comply with the instruction to perform MVCs. In order to avoid such compliance-related recording failures, a more prominent reminder, such as an audio alarm signal, could be used to stimulate participants to perform the MVCs. Moreover, an EMG scoring method that is independent of the presence of a MVC can be considered, provided, as discussed above, its validity has been established.

A final comment should be made regarding the choice of predictor variables in future studies assessing the effect of sleep bruxism on dental implants. The influence of confounding variables when interpreting the results of such studies has also been reported by Chrcanovic et al.^2^ In the current study, four variables were chosen as possible confounders, i.e., smoking status, awake bruxism, peri-implant plaque accumulation, and periodontal parameters, based on available literature.^11^ Other variables, such as implant geometrical characteristics and antagonist status were collected, but only for the purposes of a complete description of the sample. It may be argued that these parameters should also be considered as confounders. However, doing so would have significant implications for the final sample size.^16^ Moreover, careful selection of variables is also important considering that a large number of such variables can increase the risk for type I error. Given the significant number of variables that can be assessed in a bruxism–dental implant complication study (e.g.,^27^), it is suggested that future studies in the field include at least a set of ‘classic’ confounders/covariates, i.e., smoking and periodontal parameters, variables emerging from clinical studies (e.g.,^27^), and variables emerging from the experience of dentists in daily practice (e.g.,^26^).

## Conclusion

The conduct of a prospective clinical cohort study on the associations between sleep bruxism and (peri-)implant complications should take the following factors into account:

- Participant recruitment: rates can be low; a multicenter approach and/or an extensive recruitment period should be considered, based on thorough and realistic evaluations of the recruitment potential at each study site,
- sleep bruxism recordings: failures can occur as a result of low participant compliance and device detachments; EMG devices should be simple and minimally burdening in their use, and it is suggested that the quality of the raw EMG signal is evaluated, and
- the choice of predictor variables is important in terms of sample size and statistical considerations; it is suggested that it is based on the results of clinical studies.
